# Jordi Mancebo, in memoriam (August 06 2022)

**DOI:** 10.1186/s13613-022-01061-1

**Published:** 2022-09-14

**Authors:** Laurent Brochard, Alain Mercat

**Affiliations:** 1 Keenan Research Centre for Biomedical Science, Li Ka Shing Knowledge Institute, Unity Health Toronto, 209 Victoria St, Toronto, ON M5B 1T8 Canada; 2 Interdepartmental Division of Critical Care, University of Toronto, Toronto, Canada; 3 Médecine Intensive - Réanimation et Médecine Hyperbare, CHU Angers, Angers, France

*« Il y a des êtres qui justifient le monde, qui aident à vivre par leur seule présence». Albert Camus*

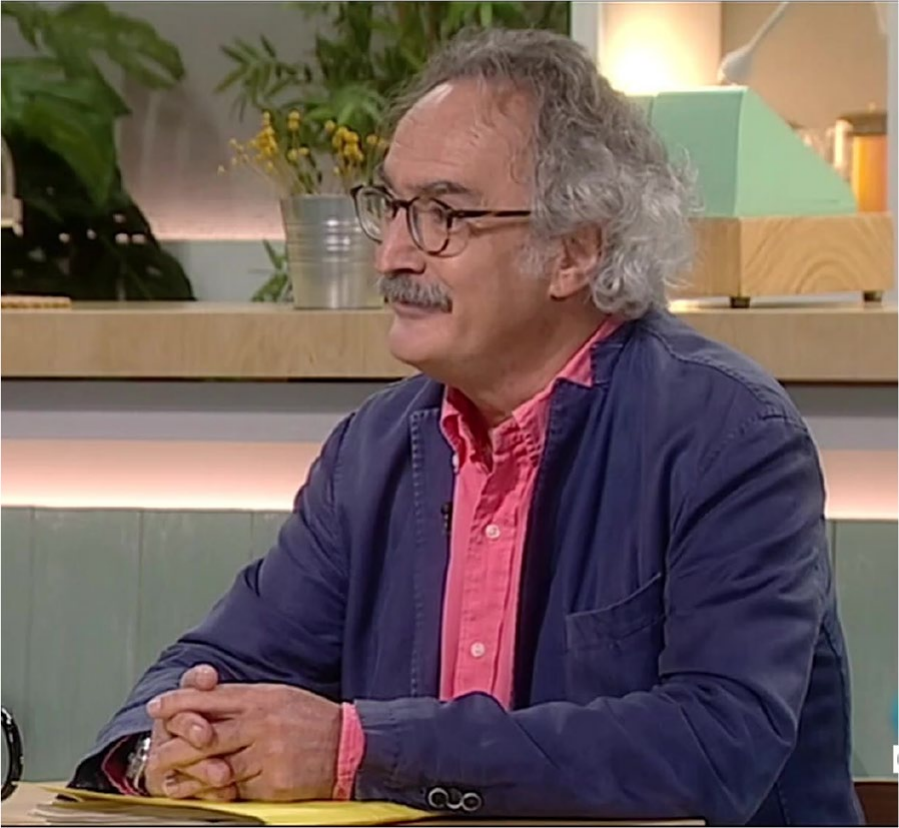
Jordi Mancebo, graduated in Medicine in 1980 and was made Doctor of Medicine in 1991 from the Autonomous University of Barcelona. He specialized in Intensive Care Medicine in 1986 and worked in Sant Pau Hospital with Alvar Net and Salvador Benito. He became the head of the ICU there. He was an outstanding clinician and mentor. Early during the COVID-19 pandemic, he started to face the challenge of managing the surge of COVID-19, which stroked quite hard in Barcelona, and the last years were very busy clinically for him, strongly supporting his colleagues and the nurses working so hard. He died peacefully at home on August 2022 with his family.

Thanks to the collaboration between Francois Lemaire and Salvador Benito, Jordi spent two periods of sabbatical in Henri Mondor Hospital in Creteil, early in his career, at the end of the 1980s and in the 1990s. He became there one of the pioneers in noninvasive ventilation participating in a scientific human and clinical adventure. From this period, he also learned to speak French (later, he was speaking five languages) and started to become very involved in the French Society of Intensive Care Medicine (SRLF), as well the European Society of Intensive Care Medicine that was still in its infancy. Jordi became naturally involved with the Spanish Society, but also the American Thoracic Society (where he was one of the few Europeans to lead the Critical Care program Committee); he also helped many programs in Portugal, and in South America to develop their academic missions. Jordi was considered as a great scientist, but very often as a true friend.

Jordi has been a great scientist, with a recognized expertise in respiratory physiology, an expert in ARDS (publishing the first RCT with almost favorable results of prone positioning) and he was one of the founder and co-lead of the Pleural Pressure Working Group. He loved the PLUG because of the interactions with the next generations of researchers and clinicians. Jordi was a dedicated teacher and he enormously liked the one-on-one interactions that he could get during seminars or courses across the world.

Jordi had convictions, he was a model of rigor in science and was always happy to defend what he believed was correct, always putting the interest of the patient first.

But anyone who met Jordi remembers his kindness, his smile, his natural way of making people feeling comfortable and his genuine interest in people.

We are sad and will miss him, and have special thoughts for his family, especially his children, Montsita and Tito, his close friends and colleagues, but we will keep in mind the figure of a man who made our life a better one.

Visit the PLUG https://www.plugwgroup.org/ where you will find multiple condolences of scientists from all over the world.

The photo comes from a television interview about the pandemic in Barcelona https://www.rtve.es/play/videos/cafe-didees/cafe-didees-dr-jordi-mancebo-a-les-uci-encara-hi-ha-400-persones/5905773/.

## Data Availability

Not applicable.

